# The Research of Conductivity and Dielectric Properties of ZnO/LDPE Composites with Different Particles Size

**DOI:** 10.3390/ma13184136

**Published:** 2020-09-17

**Authors:** Yujia Cheng, Guang Yu, Boyang Yu, Xiaohong Zhang

**Affiliations:** 1 Mechanical and Electrical Engineering Institute, University of Electronic Science and Technology of China, Zhongshan Institute, Zhongshan 528400, China; chengyujia@hrbust.edu.cn; 2 Mechanical and Electrical Engineering Institute, Zhongshan Polytechnic, Zhongshan 528400, China; 18345141862@163.com; 3 Key Laboratory of Engineering Dielectrics and Its Application, Ministry of Education, Harbin University of Science and Technology, Harbin 150080, China; xhzhang133@126.com

**Keywords:** LDPE, dispersive, electrical conductivity, breakdown properties

## Abstract

Nanocomposites exhibit a high dielectric strength, whereas microcomposites exhibit a high thermal conductivity. In this study, good insulating materials were developed on the basis of the synergetic effect of micro- and nanoparticles, which were used as inorganic fillers. With a double-melting blend, nano-ZnO/low density polyethylene (LDPE), micro-ZnO/LDPE, and micro-nano-ZnO/LDPE composites were prepared, according to the scanning electron microscope test, polarization microscope test, conductivity test, breakdown test, and dielectric spectrum test, the dielectric property of micro-nano-ZnO/LDPE was explored. The SEM test results showed that by adding a suitable proportion of ZnO particles, the inorganic particles could disperse uniformly without reuniting. The PLM test results showed that the micro- and nano-ZnO particles adding decreased the crystal size. The arrangement was regular and tight. The macroscopic results showed that the mass fraction of nanoparticles and microparticles were 3% and 2%, the samples conductivity was the lowest. The breakdown field strength of the nanocomposites increased. The breakdown field strength of nanocomposites with 1%, 3%, and 5% nanoparticle contents were 5%, 15%, and 10% higher than that of pure LDPE. The addition of inorganic particles resulted in new polarization modes: Ionic displacement polarization and interfacial polarization. The ZnO/LDPE composites exhibited a higher dielectric constant and dielectric loss factor than pure LDPE. However, with the increasing frequency, it took considerable time to attain interfacial polarization in the nanocomposite and micro-nanocomposite, thus decreasing the dielectric constant.

## 1. Introduction

With the high-voltage and extra-high-voltage applications developed, greater demands are being placed on the insulation properties of cables. Cable insulation materials can be modified by inorganic particles adding. With melting technologies, the breakdown field strength and insulation resistivity of composites can be improved. Moreover, this is the key to solving the aging of insulation cables [1,2]. Generally, in the range of 1–100 nm, these inorganic particles were the nanometer system. The size greater than or equal to 1 μm were the micrometer system. After that, in 2002, Nelson et al. reported the results of experiments conducted on nanocomposites. Since then, nanocomposites have been widely studied [3]. In 2013, Professor Frechette gave a further explanation of the concept of nanocomposites, suggesting that nanocomposites are multiphase materials containing two or more dielectrics. The multicomponent structure has at least one component with a nanostructure. This nanostructure causes a variation in one or more electrical properties [4]. A complex interface structure is formed between the polymer matrix and the inorganic particles with the addition of nanoparticles owing to their large specific surface area. The physicochemical characteristics of this interface structure significantly affect the charge transport process. The macro-electrical properties of dielectrics are also affected [5,6,7]. Nano-fillers can restrain the polymer space charges [8,9,10,11,12] and effectively improve the breakdown field strength of materials [13,14,15,16]. The insulation resistivity [17,18,19] and heat resistance can be increased as well [20,21]. The microcomposites was the composite system with one micro-size phase. The micro-nanocomposites was the composite system with both nano- and micro-size phase. Due to their large size, micro-fillers bring about a good performance in terms of thermology and corona resistance [22,23,24].

The ZnO acted as new inorganic particles, which was a kind of high performance compound semiconductor material. The band gap was 3.37 eV. Under the room temperature, the exaction binding energy was 60 meV. Therefore, the ZnO was very stable in sunlight, water, and air. Therefore, it was as one kind of multifunctional material, which was widely used in optoelectronic devices, electrochemical devices, and photochemistry devices. From the related optics research [25], the two-dimensional homogeneity nanocrystalline ZnO sheeting, of which the grain size was 20 nm, could emit the trichromatic light. The transparence curves of nanocrystalline ZnO sheeting was smooth without any interference. The composites nanostructure could obtain the excellent photocatalysis activity easily. The micron-sized was beneficial for the photocatalyst recycling. It was high efficient and environmental. In addition, the nano-ZnO particles possessed many excellent properties such as mechanics and electricity. With the nanoindentation, the hardess and elastic modulus of nano-ZnO composites could be obtained effectively. They also possessed excellent physical and chemical properties because of the regulatable nanoscale and unique form. Therefore, it has a large space for development. In addition, the nano-ZnO possessed nonlinear characteristics, the polymer is used as a matrix. Insulating materials can be prepared from nonlinear composites on the basis of the melting blend. The conductivity and dielectric constant vary with the electric field strength. Adding nano-ZnO particles can enhance the conductivity of polymers. In complex insulation structures, the electric field distributions can self-homogenize, thereby increasing the dielectric strength of the insulation structure. Adding nano-ZnO particles can also improve the tensile mechanical properties of polyethylene. The volume resistivity and AC breakdown strength of polyethylene matrix composites improved, which restrain the injection of like-charges from the electrode [26,27,28,29,30,31]. In summary, the inorganic ZnO particles possessed many excellent characteristics. Therefore, in this manuscript, the inorganic ZnO particles were used as fillers, focused on the electrical properties of ZnO particles. Based on the research of nanocomposite and microcomposite technologies, a micro-nano-ZnO/low density polyethylene (LDPE) composite was prepared with LDPE as the matrix material.

According to the SEM test, the inorganic fillers aggregation and inorganic particles size in polymers could be observed, from which the inorganic particles dispersion and agglomeration in the polymer matrix were obtained. According to the PLM test, the effect of micro- and nano-ZnO particles adding on LDPE crystallization was observed, from which the effect of non-crystalline complicated structure on electronic transport was explored. According to the AC breakdown test, conductivity test, and dielectric spectrum test, the effect of micro- and nano-ZnO particles adding on carrier mobility and space charge accumulation were explored. It was based on high-field electronic energy transfer theory and interface trap characteristics. It provided the theoretical and experiment basis for the development of high dielectric property insulating materials.

## 2. Sample Preparation and Microstructure Characteristics

### 2.1. Experimental Materials and Instruments

Table 1 lists the main equipment type and parameters of the raw materials used in the experiments.

### 2.2. Sample Preparation

The melting blend was used for composites preparation. Firstly, the inorganic particles were dealt with surface modification by the silane coupling agent. Then, the inorganic particles and LDPE were dealt with the melting blend in rotational speed rheology. After that, the master batch of nanocomposite and microcomposite with 10 wt% particles content were prepared. Secondly, with two masterbatches and pure LDPE in different blending proportions, three different ZnO composites were prepared. The samples were then pressed using a plate vulcanization press machine (model: XLB25-D). At a pressing temperature of 150 °C, pressure of 15 MPa, and pressing time of 25 min, the samples with thicknesses of 100 and 200 μm were pressed. The 200-μm-thick samples were used as conductivity samples, both sides of which were evaporated with an aluminum electrode using a three-electrode system (the diameter of the measuring and high-pressure electrodes were 50 and 74 nm, respectively. The inner and outer diameters of the guard electrode were 54 and 74 nm, respectively). The 100 μm thick samples were used as dielectric frequency spectrum samples, both sides of which were evaporated using an aluminum electrode with a diameter of 50 nm. Composites with a nanoparticle mass fraction of 1%, 3%, and 5% are denoted by N1, N3, and N5, and composites with microparticle contents of 1%, 3%, and 5% are denoted by M1, M3, and M5, respectively. The micro-nanocomposites with 1% nanoparticles and 4% microparticles, 2% nanoparticles and 3% microparticles, and 3% nanoparticles and 2% microparticles are denoted by N1M4, N2M3, and N3M2, respectively.

### 2.3. SEM Characterization

In this manuscript, the ZnO particles dispersion in the matrix were observed by SEM. From this test, the morphologies of the micro-ZnO and nano-ZnO composites could be observed, from which the dispersion of the microparticles and nanoparticles in the matrix material was explored. Firstly, these samples were placed in liquid nitrogen for brittle fracture. The microstructures of the composites were then observed.

**Figure 1:**
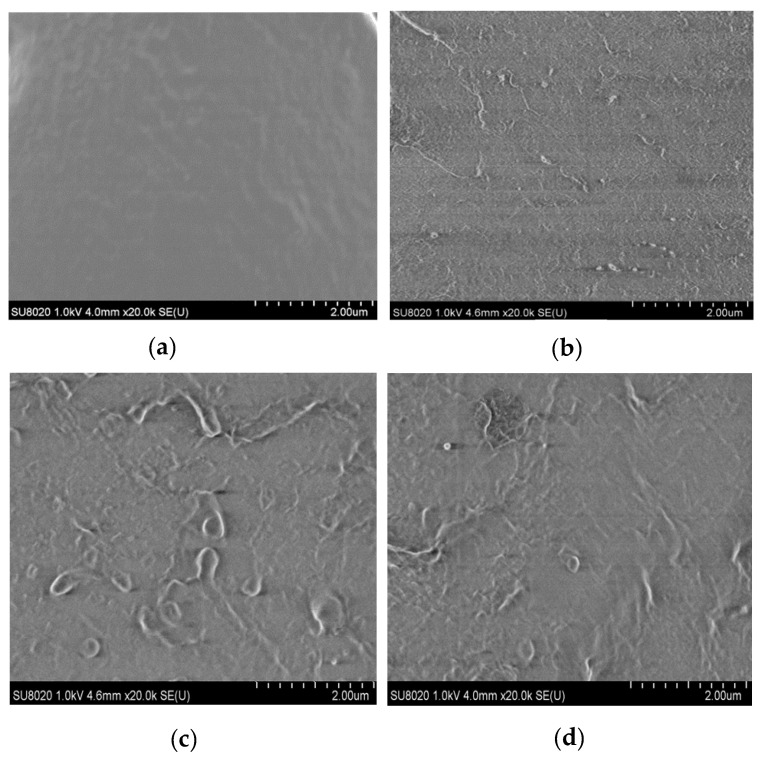
SEM patterns of (**a**) low density polyethylene (LDPE); (**b**) N5; (**c**) M5; (**d**) N3M2.

The sample containing ZnO particles with a mass fraction of 5% was used for the test. In Figure 1, the four samples were pure LDPE, nano-ZnO/LDPE with 5% nanoparticles content, micro-ZnO/LDPE with 5% microparticles content, and micro-nano-ZnO/LDPE with 2% microparticles and 3% nanoparticles content. When the additive content was high, the dispersion and reunion of the inorganic particles in the polymer matrix were explored. Figure 1 shows the SEM patterns of pure LDPE and of the different composites. In the figure, the light-colored parts represent the additive (ZnO particles), and the dark part indicates the matrix LDPE.

As shown in Figure 1b, inorganic-phase ZnO particles are small. Therefore, there is no evident agglomeration due to the addition of inorganic ZnO particles in the LDPE matrix. As shown in Figure 1c, when the microparticles are large and the mass fraction is high, the volume of the bright region (inorganic phase) inside the sample increases. Along with the SEM patterns of the samples, it can be found that the number of ZnO particles in the micro-nano-ZnO/LDPE is the highest under the same mass fraction of the inorganic particles; however, the agglomeration of the ZnO particles is not evident in the matrix. Hence, it can be concluded that the microparticles and nanoparticles were dispersed more uniformly in N3M2.

### 2.4. PLM Characterization

In order to characterize the effect of micro- and nanoparticles adding on polymers crystalline morphology, the LeicaDM2500 polarization microscope (Leica, Weztlar, Germany) was used to observe the composites. Firstly, the samples were put into the 5% concentration potassium permanganate/concentrated sulfuric acid mixed solution. After 24 h corrosion, the samples were taken out. Secondly, all the samples were cleaned in an ultrasonic machine. Then, they were placed on a glass slide and the crystallization was observed.

**Figure 2:**
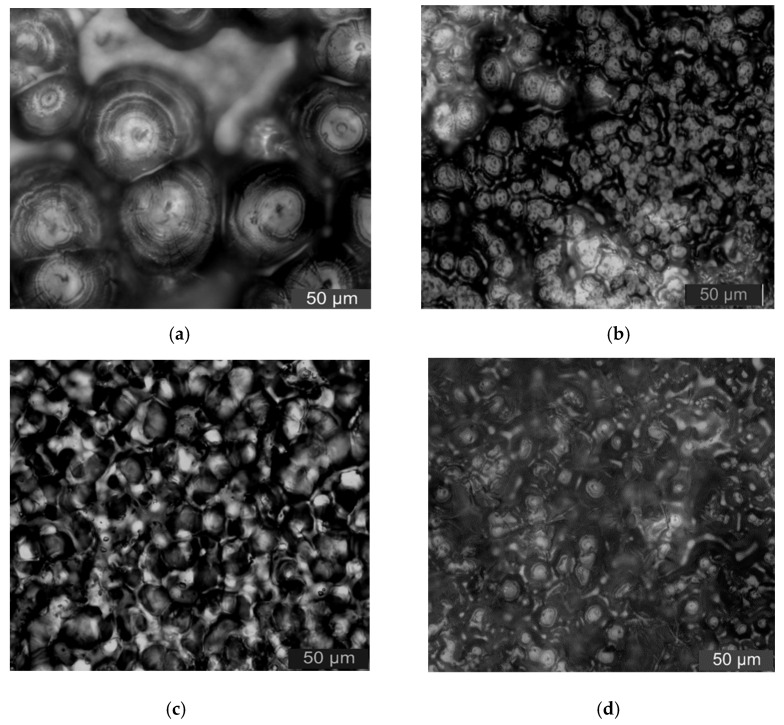
PLM patterns of different samples (**a**) LDPE; (**b**) N5; (**c**) M5; (**d**) N3M2.

The samples with 5% ZnO-particles content were used for the PLM test. In high inorganic particles of additive contents, the effect of inorganic particles adding on crystalline morphology of polymer matrix was explored. The PLM test results of different samples were shown in Figure 2.

From Figure 2, the crystal size in samples LDPE was larger. The larger non-crystalline area existed between grains, and the grains arrangement was loose. The ZnO particles adding decreased the polymers crystal size obviously. The grain arrangement was close and the interface between the spherocrystal was blurred. When the ZnO particles content was 5%, the crystal size of samples N5 and N3M2 were both higher than that of sample M5, and the gains arranged closely. The crystal size of sample N3M2 was between sample N5 and M5. This was because the ZnO particles adding was equivalent to introducing the heterogeneous nucleating agent to the matrix. The nucleation centers increased, so the crystal size of composites was smaller. The crystallization quantity, non-crystalline areas, and interface structure increased. The increasing of non-crystalline structure decreased the carrier mobility. Meanwhile, there were traps lever in the composites interface structure. Therefore, the samples conductivity decreased and the breakdown characteristics increased. In one word, the inorganic ZnO particles adding changed the composites crystal morphology. In addition, it affected the traps depth and density in polymers. In addition, the carrier mobility in the samples was also affected. Therefore, the crystal morphology of polymer composites was different, the dielectric properties were also different, in addition to the surface atoms. The specific surface area and surface energy of nano-materials were large, so the surface effect made a great effect on the nano-materials mechanical properties. Compared with the macro-scale, the mechanical property of nanomaterials was different. According to the nano-identation test, the hardness, elastic modulus, and plastic deformation of nanocomposites could be obtained in a situation of non-destructive and surface micro-damage. From the related research [32,33,34], when the interfacial bonding strength of micro-nanocomposites was low, the fiber modulus showed parabola with the increasing of pressed depth. With the same indentation depth, the indentation hardness increased with the decreasing of particles size. According to the PLM test, the nanocomposites crystalline size was small. The crystalline structure was close. The hardness was high and the mechanical properties were strong.

## 3. Results and Analysis of Macroscopic Tests

### 3.1. Breakdown Characteristics

In the AC breakdown test, the system boosted uniformly. When the samples were breakdown, the pressurizing was stop. The voltage at this moment and the thickness of breakdown points *d* were measured. Therefore, the breakdown field strength *E* could be obtained from the formula *E = U/d*. Each sample was tested 30 times. The data were analyzed by MINITAB. Finally, the Weibull distribution parameters *E* and *β* were obtained.

**Figure 3:**
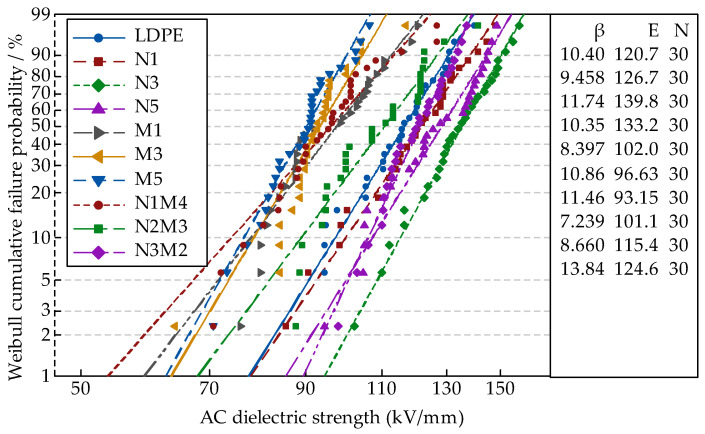
Weibull distribution curves of the micro, nano, and micro-nano ZnO/LDPE composites.

**Figure 4:**
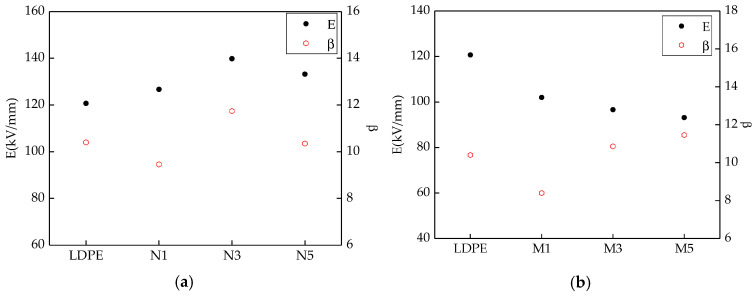

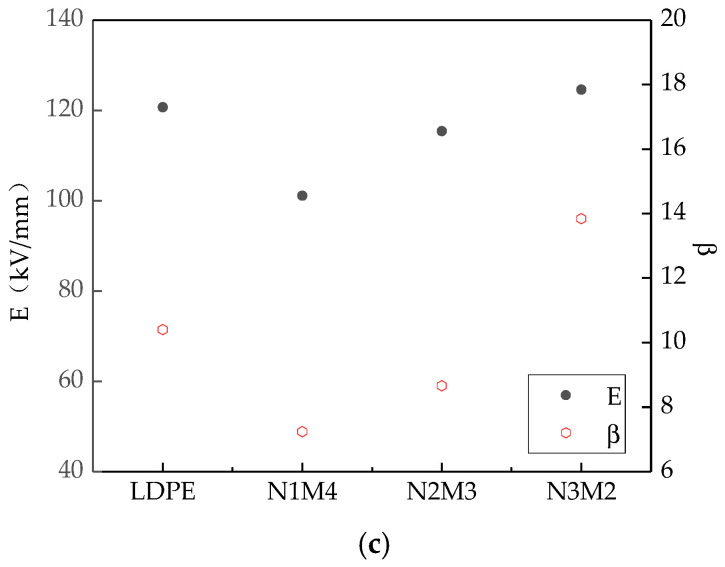
Weibull distribution parameters changes of three samples: (**a**) Nano ZnO/LDPE composites; (**b**) micro ZnO/LDPE composites; (**c**) micro-nano ZnO/LDPE composites.

Based on the previous test results [35], the effects of different ZnO particle sizes and mass fractions on the breakdown properties of the samples could be explored. Figure 3 shows the breakdown test results of the different samples. The Weibull distribution parameters changes of different samples were shown in Figure 4. Among them, the scale parameter *E* reflected the samples breakdown field strength. The shape parameter *β* reflected the breakdown voltage dispersion [36]. The bigger the value of *β*, the better the particles dispersion was.

From Figure 3 and Figure 4, with different sizes of ZnO particles adding, the breakdown field strength of composites were different. When the inorganic particle contents were 3%, the breakdown field strength was the highest 139.8 kV/mm, which was 15.8% higher than the polymers matrix. With the nanoparticles increasing, the composites breakdown field strength decreased. For the microcomposite, the breakdown field strength decreased gradually with the ZnO particles increasing. For the micro-nanocomposite, while the contents of nanoparticles and microparticles were 3% and 2%, the breakdown field strength was the highest 124.6 kV/mm. With the microparticles increasing, the composites breakdown field decreased gradually. In the analysis of the reasons, the nanoparticles possessed excellent characteristics. They would combine with the polymer matrix by a covalent bond, and the close interfacial structure was formed. The free volume of non-crystalline areas decreased. In the interfacial charge layer, the electrons would scatter. The deeper traps level would be introduced by nanoparticles adding [37], so the breakdown field of nanocomposite was the highest. With the increasing of nanoparticles, the particles distance was smaller. The transitional areas coincided and the barrier energy level decreased, from which the carrier migrated easily. At this time, the breakdown field strength of nanocomposite decreased. Owing to the weak interfacial binding force of micro-particles and matrix, the breakdown field strength of the microcomposite was lower [38]. For the micro-nanocomposite, the breakdown field strength was higher than that of the microcomposite. However, the microparticles introduced some defects, so the breakdown field strength was also lower than that of the nanocomposite.

### 3.2. Conductivity Characteristic Test

The conductivity characteristic is an important parameter characterizing the dielectric properties of materials. Based on the conductive current test conducted on the different composites, relevant data could be obtained for the engineering application of ZnO/LDPE composites. The data include the conductivity mechanism, charge injection, and transport. With an in-depth understanding of the relationship between the electrical properties and the microstructure, a theoretical basis could be provided for the development and design of new insulating materials. Therefore, a conductivity characteristic test was conducted on all the samples.

#### 3.2.1. Effects of Inorganic Particle Mass Fraction on the Electrical Conductivity of Composites

**Figure 5:**
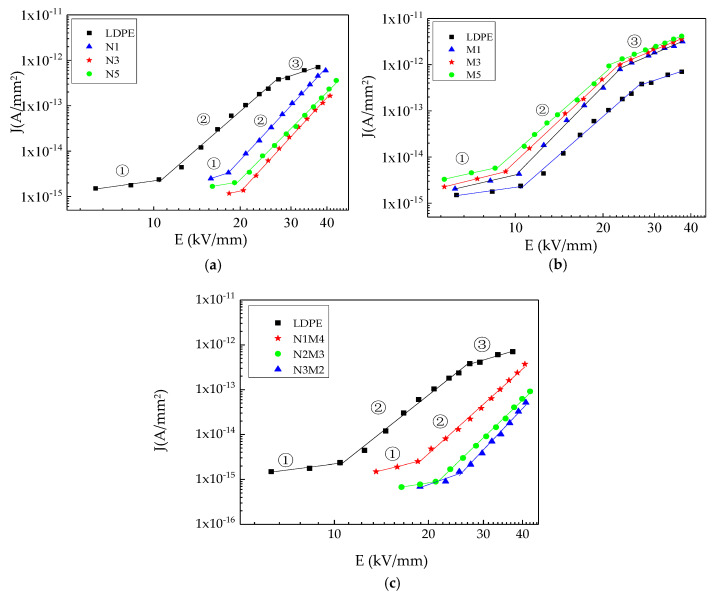
Relationship between conductivity current and field strength of different samples: (**a**) Nano ZnO/LDPE composites; (**b**) micro ZnO/LDPE composites; (**c**) micro-nano ZnO/LDPE composites.

In the conductivity experiments, a galvanometer with a measuring accuracy of 10^−14^ A was used. The thickness of the samples was 200 μm, and an aluminum three-electrode system was plated on the sample surface. With the step-boost method, each point was tested every 600 s, and all the experimental results were recorded. With the increasing applied electric field, the variations in the conductive current of the samples were plotted. Figure 5 shows the conductive current volt–Ampere characteristic curves of the samples. 

As shown in Figure 5, in the log–log coordinate system, there were three conductivity areas in the pure LDPE test range. This phenomenon coincided with the space charge limited current (SCLC) theory of solid dielectric, so the composites conductivity current could be analyzed by the SCLC theory. Ideally, there were three significantly different conductivity areas in the J-U curve of solid dielectric. In the first area, the electric field was relatively low. The charges injected by the electrode were captured by the traps, so they did not contribute to the current. The slope of current density and electric field was 1, which coincided with the four test samples in Table 2. This illustrated that the current in the first area was Ohmic current. In the second area, when the electric field exceeded E1, the charges injected by the electrodes increased rapidly. The steady-state free charges also increased, which caused the index of the electric field to be greater than 2. The current did not obey the Ohm’s law, and the slope of current density and electric field was 6. At this time, the current coincided with the SCLC theory. From the experimental results, the changing trends of the pure LDPE and micro-ZnO composite are similar in the entire electric field range. Three evident change areas appear in the conductive current curve, consistent with the results of a relevant study [39]. In contrast, only two evident change areas, namely ① and ②, appear in the conductive current curves of the nano-ZnO and micro-nano-ZnO composites. The data were fitted in the log–log coordinate, from which the slopes and transition threshold field strengths of the different ZnO/LDPE composites in the different conductivity areas were obtained. Table 2 lists the results. The slope value after fitting is in accordance with the space-charge limited current theory. The transition electric field threshold in the three areas were marked by *E*_1_ and *E*_2_.

From Table 2, the slope values of the four samples in the three conductivity areas ①, ②, and ③ are approximately 1, 5.5, and 2, respectively. The threshold field strength *E*_1_ of the nano-ZnO and micro-nano-ZnO composites increased to different extents. The threshold field strength of the micro-ZnO composites was lower than that of pure LDPE. Related studies have reported a certain relationship between the threshold field strength *E*_1_ and the trap depth [40], as expressed in Equation (1).

(1) $$E_{1}\propto exp(\frac{\Delta U}{kT})$$

In Equation (1), *E*_1_ is the threshold field strength; *k* is a constant; *T* is the absolute temperature; Δ*U* is the trap depth. Based on the threshold field strengths, the trap depth of the four composites is in the following order: Micro-nano-ZnO composites > nano-ZnO composites > LDPE > micro-ZnO composites. Due to the trapping effect on the carriers, the carrier mobility is reduced. Accordingly, the conductivity of the four samples is in the following order: Micro-nano-ZnO composites < nano-ZnO composites < LDPE < micro-ZnO composites. From the previous research, the threshold voltage *U* varied directly as the traps density. Since the threshold voltage corresponded with the threshold electric field, the threshold electric field varied directly as the traps density. Therefore, the threshold field strength *E*_2_ of the nano-ZnO and micro-nano-ZnO composites was undetectable in the range of the applied electric field. The *E*_2_ value of the micro-ZnO composites was similar to that of pure LDPE. This shows that the trap densities of the two samples are approximately the same. In conclusion, deep traps were introduced by both the nanocomposite and micro-nanocomposite. The higher trap density in the dielectric could help capture the carriers more easily. The directional migration of the electrons in the direction of the electric field was blocked, and the carrier mobility was reduced. Moreover, the probability of thermal excitation of the electrons in the deep traps was low, and only a few free electrons participated in electric conduction. Therefore, the conductivity of the micro-nano-ZnO and nano-ZnO composites was low. The trap density introduced by the microparticles was low. The introduction of more physical defects increased the number of shallow traps. Moreover, adding microparticles was equivalent to adding impurities into the dielectric. Thus, the conductivity of the micro-ZnO composites was higher than that of pure LDPE. This was consistent with relative research in some way [41]. However, in this manuscript, the different micro- and nano-ZnO/LDPE were dealt with the conductivity test under different field strengths. The effect of trap characteristics on different samples conductivity characteristics could be explored further.

#### 3.2.2. Effect of Experiment Temperature on the Conductivity Characteristics of the Composites

**Figure 6:**
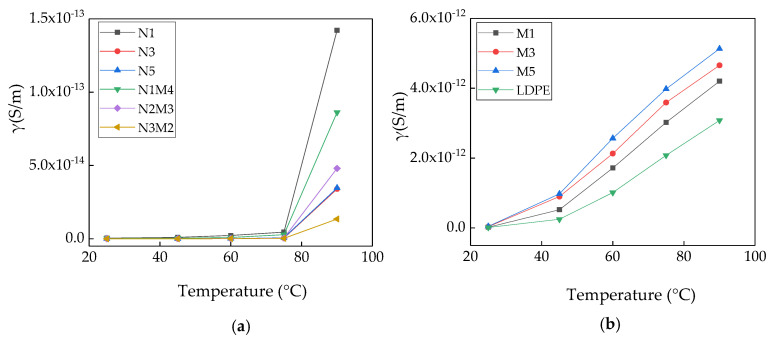
Variation in the conductivity of samples with the temperature: (**a**) Nano ZnO/LDPE and micro-nano-ZnO/LDPE samples; (**b**) LDPE and micro ZnO/LDPE samples.

To explore the effect of temperature on the conductivity of the samples, a conductivity test was carried out at a field strength of 20 kV/mm. The temperatures were set to 25, 45, 60, 75, and 90 °C, respectively. Each sample was tested at the same temperature three times. According to the calculation formula, the conductivity values of each sample were obtained, and then averaged. In all the measurements, the one closest to the average value was chosen for the final measurement of the conductivity. Figure 6 shows the variation in the conductivity of the samples with the temperature.

As shown in Figure 6a, with the increasing temperature, the conductivity of all the composites exhibit a gradually increasing trend. Across the tested temperature range, with the increasing nanoparticle mass fraction and temperature, the ascending range of the conductivity reduces. The conductivity of N1 is significantly higher than those of N3 and N5. When the experimental temperature is low, the change range of the conductivity of the nano-ZnO/LDPE with the temperature is not evident. This was because the interface charge layer between the nanoparticles and the matrix material had a scattering effect on the carrier. In the high-temperature area, the change range of the conductivity of the nano-ZnO/LDPE with the temperature is evident. For the micro-nanocomposite, the change range of the conductivity with the temperature is the same as that of the nanocomposite. The conductivity of N3M2 is the lowest in the entire temperature range. As shown in Figure 6b, with the increase in the temperature, the conductivity of the microcomposites increases gradually. At the same temperature, the conductivity increases gradually with an increase in the micro-ZnO mass fraction. Across the range of testing temperature, the change range of the conductivity of the micro-ZnO/LDPE with the temperature is evident. This shows that the conductivity of the micro-ZnO/LDPE composite strongly depends on the temperature.

The test results are shown in Figure 6a,b. The crystalline and non-crystalline regions work together in the polyethylene matrix. The non-crystalline regions break the energy band in the polymer, and the barriers between the crystalline regions are overcome in the process of carrier migration. When the temperature increases, the carrier gains higher energy. Moreover, the molecular gap in the LDPE increases, and there are fewer barriers that need to be overcome in the process of carrier migration. Therefore, the conductivity of pure LDPE was easily affected by the temperature. In contrast, shallow traps were introduced by the micro-ZnO particles in the matrix, and only weak intermolecular forces existed between the microparticles and the polythene matrix. As the temperature increases, the carriers easily migrated. Therefore, the conductivity of the micro-ZnO/LDPE was easily affected by the temperature. The additions of both nano-ZnO and micro-nano-ZnO particles introduced deep traps in the polythene matrix, which restrained the carrier migration. In conclusion, the conductivity of pure LDPE and micro-ZnO/LDPE composites had a strong dependence on the temperature; however, the conductivity values of the nano-ZnO/LDPE and micro-nano-ZnO/LDPE composites exhibited opposite trends.

**Figure 7:**
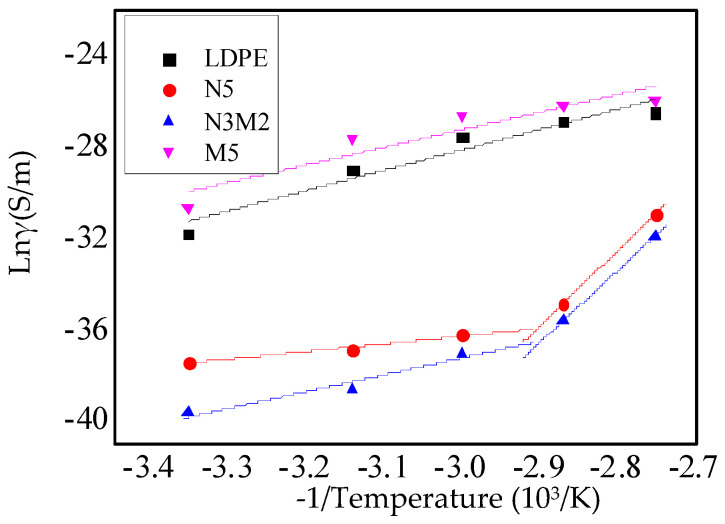
Relationship between the reciprocal of the temperature and the logarithm of the conductivity of different samples.

Figure 7 shows the relationship between the reciprocal of the temperature and the logarithm of the conductivity. The mass fraction of ZnO particles in all these composites was 5%. In Equation (2), the slope of the curve represents the activation energy.

(2) $$\ln\gamma =\ln A-\frac{B}{T}$$

As shown in Figure 7, at low temperatures, the ion activation energies of the nanocomposites and micro-nanocomposites are lower. At high temperatures, the intrinsic ionic conductivity is the dominant factor. At this time, the molecular chains in the non-crystalline area and weakly crystalline area of polyethylene begin to move, which provided the path for carrier movement. However, the carrier movement would be blocked by the nanoparticles. In addition, the nanoparticles adding introduced the traps energy levels in the composites. It was equivalent to introduce higher barrier energy levels, which strengthened the blocking effect to the electronics. The activation energy required for micro-nanocomposite dissociation increased. From the experimental result shown in Figure 7, the inflection point does not appear in the fitting curves of the micro-ZnO/LDPE composite and pure LDPE. With the increase in the temperature, the ion activation energy changed slightly. Moreover, at high temperatures, the activation energy required for micro-ZnO/LDPE dissociation decreased. This was because of the weak bonding in the microparticles and matrix. The activation energy required for microcomposite dissociation was relatively low and changed significantly at different temperatures. 

### 3.3. Dielectric Frequency Spectrum Characteristic Test

In this test, the samples thickness and diameter were 100 μm and 40 nm. The Concept 80 broadband dielectric spectra analyzer produced by Novocontrol GmbH was used for this test. The testing frequency range was 10^0^–10^5^ Hz. The 25 mm-diameter aluminium electrode system was evaporated into the upper and lower surfaces of the samples using a vacuum coating machine. To eliminate the moisture and residual charges, these samples were pretreated. Firstly, the samples were placed in an oven, maintained at a temperature of 80 °C. After the short circuit treatment for 24 h, the different samples were dealt with the dielectric frequency spectrum test under the room temperature in accordance with the GB1409-1988 standard.

**Figure 8:**
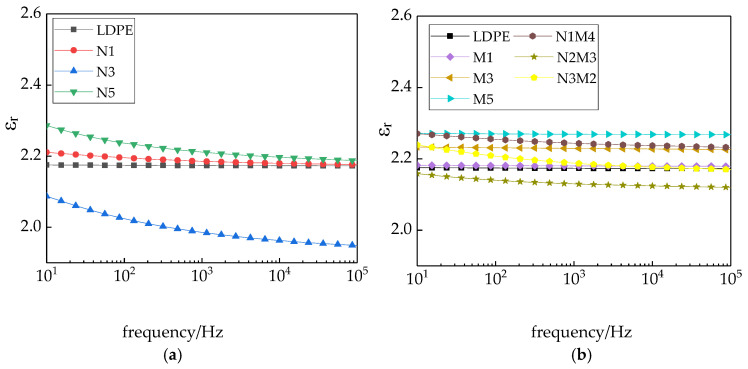
Variation in the dielectric constant of different samples with the frequency: (**a**) LDPE and nano-ZnO/LDPE samples; (**b**) micro ZnO/LDPE and micro-nano-ZnO/LDPE samples.

Figure 8 shows the variation in the dielectric constant of the different samples with the frequency.

As shown in Figure 8, in the entire test frequency range, the changes in the dielectric constants of the LDPE and micro-ZnO/LDPE composite are consistent. With the increasing test frequency, the dielectric constant slightly changes. With the increasing micro-additive concentration, the dielectric constant increases. The change trends of the nano-ZnO/LDPE and micro-nano-ZnO/LDPE composites were consistent. With the increasing test frequency, the values decrease gradually and then flatten. Among them, the dielectric constant of nanocomposite was relatively low. When the mass fraction of the nanoparticles is 3%, the relative dielectric constant of the micro-nano-ZnO/LDPE composite first decreases and then increases.

The reasons for this observation are many. The ZnO has a strong polarity. With the ZnO particles added into the polyethylene, the relative dielectric constant of the composites increased. Due to the surface effect and small size effect of the nano-ZnO particles, they reacted with the polyethylene molecule chains, forming crosslinking points. The interaction between the molecular chains was strengthened, which brought the internal structures of the polymer closer. The movement of the molecular chains was restricted. Therefore, the orientation polarization of the materials was difficult to attain. Based on the formation mechanism of the nanocomposite interface, the two-phase establishing the intrinsic polarization was limited by the nanocomposites interface. In the two phases, the center of unlike charges adsorbed each other on the surface by electrostatic forces. The strength based on the electric attraction absorption was much higher than the electric field torque, which was the electric field applied to the dipole. The intrinsic relaxation polarization was difficult to establish with the effect of applied electric field, which reduced the dielectric constant of the nanocomposites [42]. In summary, the nano-ZnO particles reduced the dielectric constant of the polymer composites. When the nanoparticle content was low, the extent of the interface formed between the nano-additives and the polyethylene matrix was less. The movement of the molecular chains in the polyethylene material was slightly restricted. The reduction range of the dielectric constants was less. With the increasing nanoparticle content, the interface formed between the nano-additive and the polyethylene matrix widened. The movement of the molecular chains of polyethylene was significantly restricted, and the dielectric constant decreased further. With a further increase in the nanoparticle content, the nanoparticles agglomerated, which decreased the interface formed between the nanoparticles and the matrix. Although there was less restriction of the molecular chain movement, the dielectric constant increased. Moreover, an interfacial polarization existed in the nanocomposite and micro-nanocomposite. The time required to attain this polarization was considerable. With the increasing test frequency, the relaxation polarization could hardly be attained. Therefore, the relative dielectric constant of the composites decreased with the increasing frequency increasing and then leveled off. In the microcomposite, only weaker physical actions existed between the micro-ZnO particles and the LDPE molecular chains. No closed interface structure was formed. Therefore, the dielectric constant of the microcomposite hardly changed with the increasing frequency. This was consistent with the results of pure LDPE. This further demonstrates that micro-ZnO particles served as a type of common additive agent in the polyethylene matrix. The particles did not closely bond with the polyethylene matrix.

**Figure 9:**
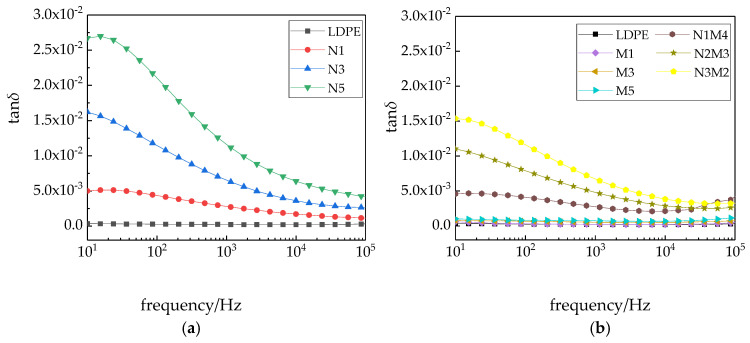
Variation in the dissipation factors of different samples with the frequency: (**a**) LDPE and nano-ZnO/LDPE samples; (**b**) micro ZnO/LDPE and micro-nano-ZnO/LDPE samples.

Figure 9 shows the variation in the dissipation factors of the different samples with the frequency. 

As shown in Figure 9, in the entire testing frequency range, the dissipation factors of the different composites are higher than that of pure LDPE. When the applied frequency increased, the dissipation factors of the nanocomposites and micro-nano composites exhibit a decreasing trend, before decreasing to a relatively stable value [43]. The main reason, in the low-frequency region, *ωτ* was less than or equal to 1, and the dissipation of the composites was due to the conductive loss. While the *ω* value tended to 0, the dissipation factor tended to infinity. The changes in the dissipation factor of the micro-ZnO/LDPE and pure LDPE composites were consistent and changed little with the increasing frequency. An interface structure was introduced with the addition of nanoparticles, which led to interface polarization in the composites. A dipole moment was formed, and the dissipation factor of the composites increased. With the increasing test frequency, the interface polarization was difficult to establish. Therefore, the dielectric loss due to the interface polarization reduced, and the dissipation factors of the nano-ZnO/LDPE and micro-nano-ZnO/LDPE composites decreased with increasing frequency.

## 4. Conclusions

The following conclusions can be drawn from the test results:

(1) From the PLM test result, the inorganic ZnO particles doping played the role of nucleating agent, which made the crystal size decrease and crystal nucleus quantity increase in polymer composites. In addition, the non-crystalline region interface structure in the samples increased, and the grains arrangement was regular and close in dielectric. From the test result of SEM, the nanoparticles and microparticles after surface modification dispersed uniformly in the polymer, which avoided the agglomeration effectively.

(2) Adding microparticles and nanoparticles had different contributions to the breakdown field strength of pure LDPE. The breakdown field strength of the nano-ZnO/LDPE nanocomposite was the highest, 15.82% higher than that of pure LDPE. The breakdown field strength of microcomposite was the lowest. The breakdown field strength of micro-nanocomposite was between that of microcomposite and nanocomposite. The breakdown field strength of the micro-nano-ZnO/LDPE composite was 3.23% higher than that of pure LDPE. However, under the same mass fraction of different ZnO particles, the shape parameter β of micro-nano-ZnO/LDPE was the highest. This showed that the inorganic particles dispersed uniformly in the polyethylene matrix with the addition of microparticles, and the agglomeration of the nanoparticles reduced.

(3) The conductivity test results showed that the conductivity of the samples was in the following order: Micro-nano-ZnO composites < nano-ZnO composites < LDPE < micro-ZnO composites. Moreover, the relationship between the conductive current and the field strength was in accordance with the space-charge limited current theory. The deep traps were introduced into the nanocomposite and micro-nanocomposite, and the traps density was high. Therefore, the carrier was captured effectively. The electrons directional migration toward the electric field was blocked, which reduced the carrier mobility. The shallow traps were introduced by microparticles. In addition, these particles were introduced into the polymer as impurity ions. Under the effect of applied electric field, the composites conductivity rose due to the impurity ions ionization. The reasons for the degradation in the carrier concentration and mobility were explained on the basis of the localized state theory.

(4) The addition of inorganic ZnO particles changed the relative dielectric constant of the composites. The ZnO particles exhibited a high dielectric constant, and an interfacial polarization was introduced. Therefore, the dielectric constant of the ZnO/LDPE composites was higher than that of pure LDPE. With increasing frequency, the relaxation polarization was difficult to attain, and the dielectric constant of the composites decreased, the surface crystalline structure order of nano-ZnO/LDPE restrained the movement of polymer macromolecule chains effectively. Therefore, the dielectric constant of nano-ZnO/LDPE was lower than that of pure LDPE, while the interfacial polarization caused a dipole moment. Therefore, the dielectric loss of the composites was higher than that of pure LDPE.

## Figures and Tables

**Table 1:** Experimental materials and instruments.

Names	Parameters/Models	Manufacturer
LDPE	Density: 0.910–0.925 mg/cm^3^	Daqing Petrochemical Co., Ltd. (Daqing, China)
ZnO	Particle sizes: 30 nm, 1 µm	Beijing Deke Daojin Science and Technology and Technology Co., Ltd. (Beijing, China)
Torque rheometer	RM-200A	Harbin Hapro Electrical Technology Co., Ltd. (Harbin, China)
Plate vulcanization press machine	XLB25-D	Haimen Jinma Rubber and Plastics Machinery Technology Co., Ltd. (Nantong, China)
Electronic balance	YP202N	Shanghai Precision and Scientific Instrument Corporation (Shanghai, China)
Vacuum drying oven	DZF-6020MBE	Shanghai Boxun industrial Co., Ltd. (Shanghai, China)
Scanning electron microscope	S-4800	Hitachi Limited (Tokyo, Japan)

**Table 2:** Slope and transition electric field characteristics of different samples.

Samples	Slope			*E* (kV/mm)	
	①	②	③	*E_1_*	*E_2_*
LDPE	0.89	5.52	2.10	10.43	27.13
N1	2.08	6.81	–	18.20	–
N3	1.39	6.91	–	20.50	–
N5	1.06	6.38	–	19.10	–
M1	1.46	6.24	2.56	10.30	25.06
M3	1.56	5.91	2.60	9.27	22.87
M5	1.39	5.59	2.51	8.53	20.94
N1M4	1.69	6.28	–	18.50	–
N2M3	1.10	6.69	–	21.10	–
N3M2	1.37	6.93	–	22.70	–
